# Insight into the ten-penny problem: guiding search by constraints and maximization

**DOI:** 10.1007/s00426-016-0800-3

**Published:** 2016-09-03

**Authors:** Michael Öllinger, Anna Fedor, Svenja Brodt, Eörs Szathmáry

**Affiliations:** 1 Parmenides Center for the Study of Thinking, Kirchplatz 1, 82049 Pullach, Germany; 2 Psychological Department, Ludwig-Maximilians-University, Pullach, Germany; 3 MTA-ELTE Theoretical Biology and Evolutionary Ecology Research Group, Biological Institute, Eötvös University, Budapest, Hungary; 4 Institute for Medical Psychology and Behavioural Neurobiology, University Tübingen, Tübingen, Germany; 5 Parmenides Center for the Conceptual Foundations of Science, Pullach, Germany

## Abstract

For a long time, insight problem solving has been either understood as nothing special or as a particular class of problem solving. The first view implicates the necessity to find efficient heuristics that restrict the search space, the second, the necessity to overcome self-imposed constraints. Recently, promising hybrid cognitive models attempt to merge both approaches. In this vein, we were interested in the interplay of constraints and heuristic search, when problem solvers were asked to solve a difficult multi-step problem, the ten-penny problem. In three experimental groups and one control group (*N* = 4 × 30) we aimed at revealing, what constraints drive problem difficulty in this problem, and how relaxing constraints, and providing an efficient search criterion facilitates the solution. We also investigated how the search behavior of successful problem solvers and non-solvers differ. We found that relaxing constraints was necessary but not sufficient to solve the problem. Without efficient heuristics that facilitate the restriction of the search space, and testing the progress of the problem solving process, the relaxation of constraints was not effective. Relaxing constraints and applying the search criterion are both necessary to effectively increase solution rates. We also found that successful solvers showed promising moves earlier and had a higher maximization and variation rate across solution attempts. We propose that this finding sheds light on how different strategies contribute to solving difficult problems. Finally, we speculate about the implications of our findings for insight problem solving.

## Introduction

Having an insight when solving a difficult problem can be characterized as a moment of full comprehension of a solution (Sternberg, & Davidson, 1995). Understanding the underlying cognitive processes of this phenomenon seems to be a promising way to learn more about the foundations of creative, innovative, out-of-the-box thinking (Dietrich, & Kanso, 2010; Gardner, 1978; Perkins, 1981). Our study sheds light on the importance of search processes and relaxation of constraints when solving a difficult problem.

### Search and constraints

**Fig. 1:**
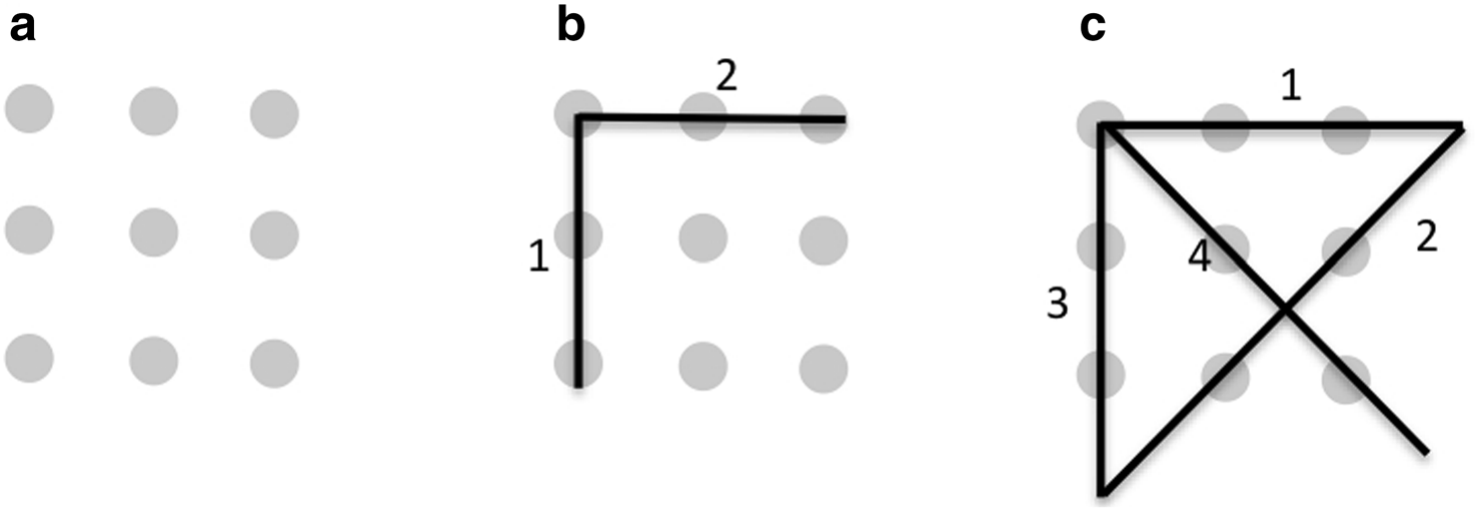
**a** The initial configuration of the nine-dot problem. **b** A solution attempt with the first two moves that connect five dots. The *numbers* indicate the sequence of moves. **c** A solution of the nine-dot problem

Two cognitive theories try to explain insight problem solving: the “nothing special” approach and the representational change theory. Kaplan and Simon (1990) assumed that insight problems are nothing special (see Öllinger, & Knoblich, 2009; Sternberg, & Davidson, 1995): they are like other problems, and it is only their huge or ill-defined search space that makes them difficult to solve. Often an exhaustive search is impossible, so the problem solver has to find the right heuristics to attain the solution. Kaplan and Simon (1990) demonstrated for the mutilated checkerboard problem that search can be facilitated by increasing the saliency of crucial problem features, which in turn increases the solution rate. Based on this idea, MacGregor, Ormerod and Chronicle (2001) built a computational model for the nine-dot problem, probably the most well-known insight problem (Burnham, & Davis, 1969; Chronicle, Ormerod, & MacGregor, 2001; Kershaw, & Ohlsson, 2004; Lung, & Dominowski, 1985; MacGregor et al., 2001; Maier, 1930). The task is to connect nine dots, arranged in a 3 × 3 matrix, by four connected straight lines (Fig. 1). MacGregor et al. postulated that two heuristics are crucial for the solution: the *maximization heuristic* states that each move should connect as many dots as possible; the *progress monitoring heuristic* tests the ratio of remaining moves and unconnected dots after each move. The authors suggested that the higher the mental look-ahead value, the more likely problem solvers realize that the problem space needs to be extended. For instance, a person with a look-ahead value of two may plan to connect three dots with the first move and two dots with the second move. As can be seen in Fig. 1b, it is impossible to connect the remaining four dots with the remaining two straight lines. MacGregor et al. (2001) assumed that this might be the moment, when the problem solver starts looking for new and “promising” moves, like drawing lines outside the given 3 × 3 grid (see Fig. 1c). A person with a look-ahead value of one would need to draw one more line before realizing that the solution is impossible.

All in all, heuristics explain how people restrict the search space and realize that the current search space is not sufficient, but they do not explain how people come up with the new search space.

Ohlsson (1992, 2011) proposed the representational change theory (RCT), which provides a mechanism exactly for that. The idea is that prior knowledge constraints or perceptual groupings are changed by mechanisms like constraint relaxation (Isaak, & Just, 1995) or chunk decomposition (Knoblich, Ohlsson, Haider, & Rhenius, 1999). In more detail, it is supposed that activation of information in the working memory alters the related long-term memory activation pattern, and eventually might help to realize previously unrecognized knowledge elements or actions (Ohlsson 1992, 2011 Chaps. 3–5). For example, in the nine-dot problem, people start with an overconstrained search space, and keep their lines inside the 3 × 3 grid, where they fail repeatedly. A representational change resolves the perceptual grouping so that problem solvers can draw lines outside the grid, to non-dot locations (see Fig. 1c). The representational change nicely explains how a problem representation and so the resulting search space is changed.

However, to equal insight with relaxing a single source of difficulty could be misleading (Scheerer, 1963; Weisberg, & Alba, 1981a, b). It was demonstrated that telling people about the main source of difficulty either directly or via transfer tasks does not necessarily increase the solution rate (Kershaw, Flynn, & Gordon, 2013; Lung, & Dominowski, 1985; Öllinger, Jones, & Knoblich, 2014; Weisberg, & Alba, 1981a, b). A few theoretical accounts combined heuristics with the representational change theory, and avoided the single cause of difficulty assumption (Kershaw, & Ohlsson, 2004; Ohlsson, 1984, 1992, 2011). Jones (2003) investigated the interplay between heuristic search and representational change. He showed that both are necessary for insight problem solving, and that they can be differentiated by move selection and eye-movement patterns (see also Knoblich, Öllinger, & Spivey, 2005, for a review). Kershaw et al. (2013) and Kershaw and Ohlsson (2004) demonstrated that prior knowledge, processing of problem information, and perceptual aspects of the problem are multiple causes of difficulty. Recently, Öllinger et al. (2014) suggested that insight problem solving could be characterized by stages of search and representational change. They proposed that in the nine-dot problem, search plays the dominant role initially. At this stage—as MacGregor et al. (2001) convincingly showed—participants rely on a maximization heuristic, i.e., they try to connect as many dots as possible with each line. After repeated failures, successful solvers change the problem representation that results in an expanded search space. Importantly, this new search space is much larger and has to be restricted by heuristics too, to guide the search. This might explain, why relaxing a constraint by cues (Weisberg, & Alba, 1981a, b) without having the right heuristics (MacGregor et al., 2001) fails, as well as why heuristics fail, if they are applied at the inappropriate search space (Öllinger et al., 2014).

### The current study

**Fig. 2:**
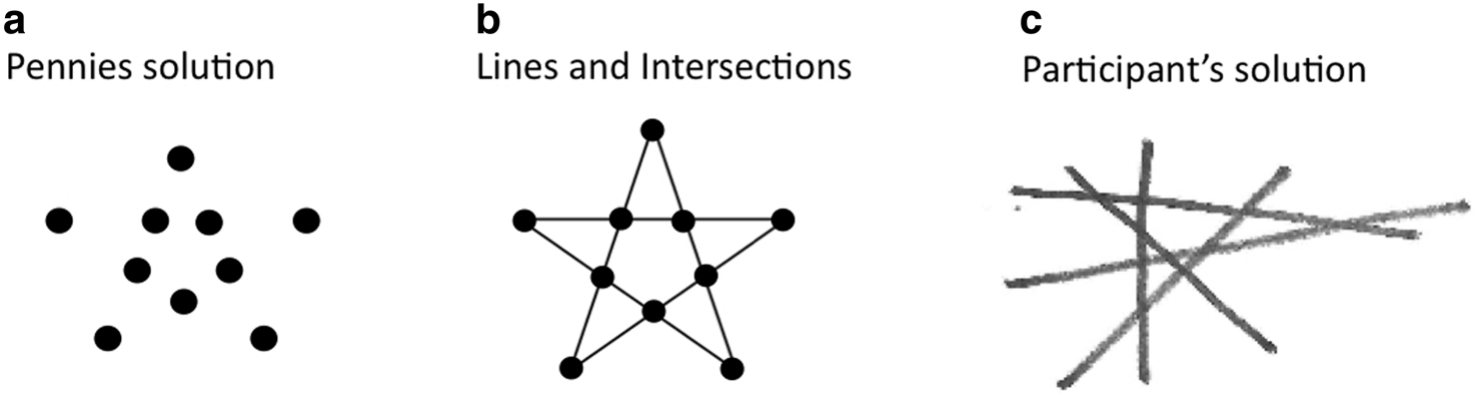
**a** Solution of the ten-penny problem with pennies. **b** Solution of the ten-penny problem using lines, intersections, and pennies. **c** A typical solution of the problem by one of our participants

We investigated how constraint relaxation and an appropriate maximization criterion might drive the problem solving process and how successful problem solvers differ from unsuccessful problem solvers. To investigate these questions, we used the ten-penny problem (Dow, & Mayer, 2004). The instruction is: “Show how you can arrange ten pennies so that you have five rows of four pennies in each row”. The solution of the problem is shown in Fig. 2a. The figure might seem confusing until lines are drawn between the dots to form a pentagram (Fig. 2b).

In the nine-dot problem, search and maximization are important both before and after the representational change (Kershaw, & Ohlsson, 2004; Öllinger et al., 2014). Before the representational change the search space is restricted by the perceptual constraint but it is still large (see also Kershaw, & Ohlsson, 2004), so it takes some time before the problem solver realizes that it is impossible to solve the problem within the perceptual boundaries. After the representational change, the search space becomes unrestricted, in fact, infinite, since the problem solver realizes that she can draw lines to non-dot points. At this point, a maximization heuristic plays an important role to direct search (MacGregor et al., 2001), and a large spatial working memory capacity is beneficial for solving the problem (Chein, Weisberg, Streeter, & Kwok, 2010).

In contrast, for the ten-penny problem, the search space is infinite from the beginning, and it might overstrain the working memory to manipulate ten single items. Manipulating pennies seems to be a constraint that problem solvers have to overcome to realize that they can manipulate the five lines (along which they arrange the pennies) instead. Paradoxically, relaxing the pennies constraint restricts the search space (Kaplan, & Simon, 1990; Knoblich et al., 1999; Ohlsson, 1992, see below), whereas in the nine-dot problem constraint relaxation expands the search space. Using lines is also a precondition for applying an efficient maximization criterion: to maximize the intersections of lines (MacGregor et al., 2001). All in all, both multi-step problems require the concerted interplay of representational change and heuristic search, but these factors might have different importance within the problem solving process of the two problems.

Hypotheses: We predict that the difficulty of the ten-penny problem is caused by the following two constraints:

1. Pennies constraint: problem solvers start out with the assumption that they have to manipulate single pennies. When they overcome this constraint, they start using lines (rows of pennies) to arrange the pennies, which reduces the complexity of the task.
2. Separate rows constraint: problem solvers assume that pennies have to be arranged in separate rows, i.e., rows do not intersect. The key to the solution is to use intersecting rows where the pennies at the intersections are part of more than one row.

There could be another constraint that problem solvers assume that pennies have to be of equal distance to each other. We think that this is only a minor constraint and left it out of consideration in this study.

We also predict that besides these constraints there is also an important process factor (Kershaw, & Ohlsson, 2004). One has to draw lines so that the number of intersections with the already drawn lines is maximal (equals to the number of already drawn lines). Each line has to intersect with all other lines, so each line has to have exactly four intersections (see Fig. 2, maximization criterion).

Consequently, we hypothesized that to solve the ten-penny problem, one has to overcome the hypothesized constraints and has to apply a maximization criterion. Thus, we predicted that providing hints about these factors would increase solution rate.

To test our hypotheses, we introduced three experimental groups and a control group. In the line group (LG), participants received a hint that using lines instead of single pennies would help to solve the problem. In the intersection group (IG), participants were informed additionally that intersections of lines would help to solve the problem. In the maximization group (MG) problem solvers received the hints about lines and intersections and were also informed that each line should have four intersections. The control group (CG) did not receive any hints.

We predicted that the solution rate of all experimental groups would benefit from the provided hints. We expected the following rank order for the solution rate MG > IG > LG > CG. We predicted a solution rate near 100 % in the maximization group.

A second aim of our study was to investigate how successful problem solvers differ from unsuccessful ones. We hypothesized, as an extension of MacGregor et al. (2001) heuristic approach, that successful solvers would show a *higher variation* in their solution attempts. This means that successful problem solving behavior is characterized using different strategies, and repeating solution attempts less frequently. According to MacGregor et al. successful solvers realize the necessity of searching for new problem states, if the initial approach fails to attain the criterion. That is, solvers would outperform non-solvers in the variance of their applied moves. Our argumentation is in close vicinity to the notion of mental set (Birch, & Rabinowitz, 1951; Chi, & Snyder, 2011; Lovett, & Anderson, 1996; Luchins, & Luchins, 1994; Öllinger, Jones, & Knoblich, 2008; Werner, & Raab, 2013). Mental set occurs when the repeated activation of a solution procedure increases the likelihood of selecting this very procedure in the future, irrespective of the existence of simpler, more efficient alternative strategies (Lovett, & Anderson, 1996), thus hindering the solution of the problem. We assumed that variation, as the counterpart of repetition, would correlate with solution rate.

## Methods

### Participants

120 participants, 42 male, were recruited via e-mails, and flyers at local universities of Munich. Most of them were students receiving course credit for participating in psychological studies, others were paid 7€. The median age was 25 years (range 18–58). The four groups did not differ with respect to age according to a one-way ANOVA, *F*(3, 112) = .59; *p* = .62. Participants were randomly assigned to either the control group or to one of the experimental groups (line group, intersection group, or maximization group).

## Materials

Participants received white, blank DIN A4 papers to draw their solution attempts.

### Procedure

Participants were tested individually in a quiet room. They gave informed consent. They received the following written instructions (in German).

*Imagine you have ten pennies. Arrange the pennies so that you have five rows of four pennies in each row.*

*Please use paper and pencil to draw your solution attempts. Please number your solution attempts consecutively.*

The time limit was 15 min. In the three experimental groups, participants received hints after 5 min. The line group received the following hint: *For solving the problem, it could be helpful to use lines instead of pennies.* The intersection group received the following hint: *For solving the problem, it could be helpful to use lines instead of pennies, and regard the intersections of lines as places where pennies rest*. The maximization group received the following hint: *For solving the problem, it could be helpful to regard the intersections of lines as places where pennies rest. Please try to draw five lines, so that each line has exactly four intersections with the rest of the lines.*

### Design

The between-subject factor was *group* and the dependent measures were solution rate, the proportion of different strategies, maximization score and variation score before and after the hint (see the definition of these variables later) (Table 1).

**Table 1:** Design

Group	0–5 min	5–15 min
Control group (CG)	No hint	No hint
Line group (LG)	No hint	“Use lines”
Intersection group (IG)	No hint	“Use lines and intersections”
Maximization group (MG)	No hint	“Maximize the number of intersections”

Except CG all groups were provided with hints after 5 min

### Data analysis

#### Strategies

**Fig. 3:**
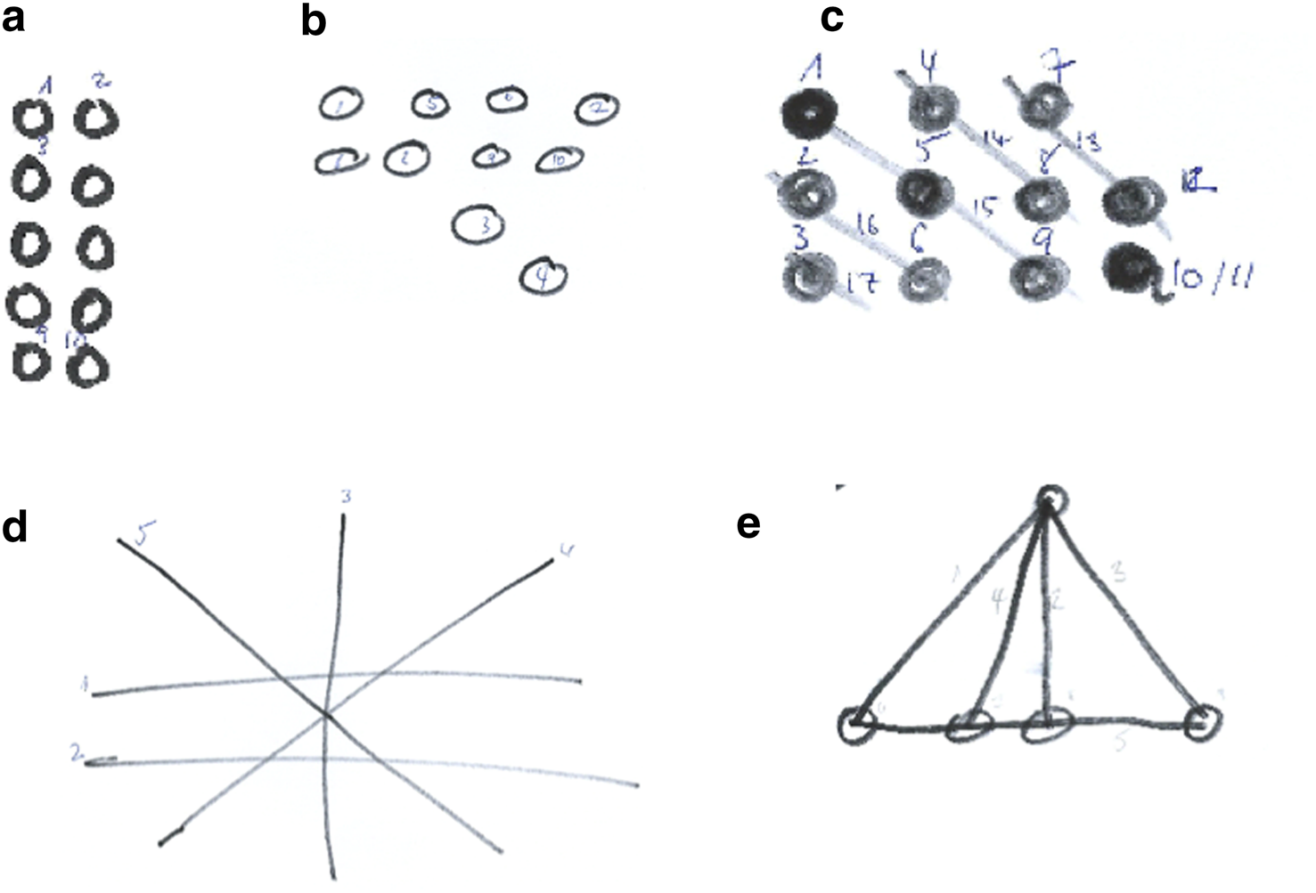
Examples for different strategies: **a**, **b** penny strategy, **c** line strategy, **d** intersection strategy with seven intersections, **e** intersection strategy with five intersections

According to our hypotheses, we defined three strategies (see examples in Fig. 3):

- Penny strategy: there are no lines in the figure, just circles or dots (see Fig. 3a, b).
- Line strategy: there are lines in the figure, but they do not intersect. Pennies are either represented in the figure or not (Fig. 3c).
- Intersection strategy: there are intersecting lines in the figure. Intersections could be in the middle of lines (Fig. 3d) or at the end points (Fig. 3e); they could be at any angle. Pennies are either represented in the figure or not.

We identified one strategy for each figure (solution attempt) that the participants drew. For figures identified with intersection strategy, we counted the number of intersections (for figures identified with any other strategy, this value was zero per definition). The experimenter marked which figures were drawn before and after the hint.

31 attempts (9 in CG, 17 in LG, 4 in IG, and 1 in the MG) out of 1229 total attempts (2.5 %) were not classifiable (e.g., the participants drew written statements or formulas instead of drawings) and were excluded. When using the intersection strategy, sometimes problem solvers used more than five lines (in 94 solution attempts out of the total 1198). We classified these attempts as intersection strategy, but we did not count the number of intersections, because the number could be higher than 10. We treated these cases as missing data for the number of intersections.

Since we were interested in the effect of hints, we excluded those participants who solved the task before 5 min elapsed (four participants).

#### Maximization score

To compare the search behavior of solvers and non-solvers, we operationalized maximization behavior by applying the following scoring system. Each solution attempt was scored +1, −1 or 0, depending on the order of the solution attempts and their strategies. The first attempt was scored 0 if it was a penny strategy and it was scored +1 if it was a line strategy or an intersection strategy. Then, each consecutive solution attempt was scored +1 if it had a “higher order” strategy than the previous one, 0 if it had the same strategy, and −1 if it had a “lower order” strategy. The order of strategies was penny strategy < line strategy < intersection strategy. Intersection strategies with more intersections were considered “higher order” than intersection strategies with less intersections. Then, we calculated the maximization score by averaging the scores separately for the first 5 min and for 5–15 min. The maximization score hypothetically ranges from −1 to +1.

Here, we provide a hypothetical sequence of strategies after a hint to illustrate the scoring (penny strategy = ps; line strategy = ls; intersection strategy = is): hint → ps → ps → ls → ps → is (three intersections) → is (1 intersection) → is (eight intersections) → is (10 intersections). Scores: ps (0), ps (0), ls (1), ps (−1), is (1), is (−1), is (1), is (1). Maximization score after hint = 2/8 = 0.25.

#### Variation rate

As a measure of variation we defined the number of +1 scores and −1 scores (see above) as change, both indicating changes in search strategy, and 0 as repetition, and then normalized it with the number of solution attempts. A number of 0 would mean that the participant used the penny strategy all the time. A number of 1 would indicate that the participant started with the line or the intersection strategy and changed strategies in each consecutive solution attempt.

#### Number of attempts

We compared the number of solution attempts (figures drawn) between solvers and non-solvers.

## Results

For our analyses, we used a 5 % significance level and all our statistical tests were two tailed. For nominal data, we used *χ* ^2^ tests and estimated the effect size by *Φ* coefficient. The data analysis on selected strategies, maximization scores, variation scores and solution attempts were evaluated by separate one-way ANOVAs before and after hints. Additionally, a reviewer of an earlier version of this manuscript suggested to calculate the differences in these measures between after and before the hint to estimate the amount of change induced by the hint and to compare the groups based on these values. For ANOVAs, we reported *η*_*p*_^2^ for estimating the effect size. For *t* tests, we determined Cohen’s *d* as effect size.

We divided the results section into three parts. First, we report results that serve as evidence for the hypothesized sources of problem difficulty. Second, we analyzed the impact of hints on problem solving behavior, finally we provide a binary logistic regression analysis assessing the influence of different predictors on the solution rate.

### Sources of difficulty

#### Pennies strategy

We controlled whether groups differed in the usage of the pennies strategy before 5 min. An ANOVA of the groups (CG, LG, IG, MG) revealed no statistically significant effect, *F*(3, 115) = .74, *p* > .52, *η*_*p*_^2^ = .02. Post hoc comparisons revealed no significant differences between the groups. Table 2 shows the number of participants who had a variation rate of zero, i.e., who used the pennies strategy exclusively. Before 5 min elapsed, there were a high number of participants with 0 variation rate. We did not find significant differences by pairwise *χ* ^*2*^ tests (*p*s > .40) between groups. After receiving hints, almost all participants dropped the pennies strategy. Pairwise *χ* ^*2*^ tests (Fisher’s exact test) showed significant differences between the CG and all other groups: CG and LG, *χ* ^2^(1, 59) = 8.68, *p* < .003, *Φ* = .38, CG and IG, *χ* ^2^(1, 58) = 8.68, *p* < .02, *Φ* = .32, and CG and MG, *χ* ^2^(1, 59) = 11.64, *p* < .001, *Φ* = .44.

**Table 2:** Number of participants who had a variation rate of zero

Group	Before 5 min	After 5 min
Control group	18/30 (60 %)	10 (33 %)
Line group	17/29 (59 %)	1 (3 %)
Intersection group	13/28 (46 %)	2 (7 %)
Maximization group	17/29 (59 %)	0 (0 %)

#### Intersection strategy

**Fig. 4:**
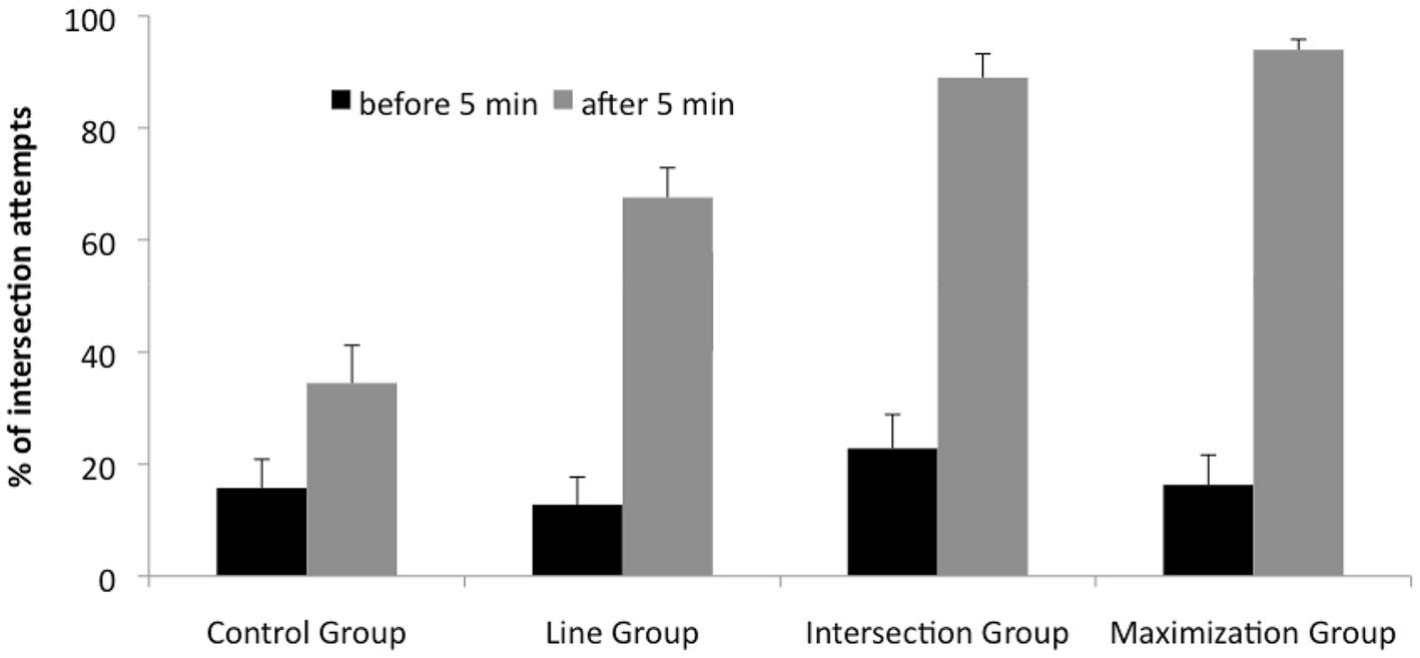
Percentage of solution attempts categorized as intersection strategy across groups. *Black bars* intersection attempts before 5 min, *gray bars* intersection attempts after 5 min. At 5 min, a hint was provided for the experimental groups. The *bars* represent the average value; the *whiskers* represent SE

We checked whether the hints affected the frequency of solution attempts with intersection strategy. For each participant, we calculated the percentage of solution attempts that was categorized as intersection strategy, separately for the solution attempts before and after 5 min (see Fig. 4). Before 5 min, the percentage of solution attempts categorized as intersection strategy was quite low in all groups. After the hint, it increased, especially in the intersection group and the maximization group.

Before 5 min, a one-way ANOVA with the between-subject factor groups (CG, LG, IG, MG) revealed no statistically significant effect, *F*(3, 115) = .82, *p* > .60, *η*_*p*_^2^ = .02. After 5 min, the percentage of solution attempts categorized as intersection strategy varied with the experimental condition. A one-way ANOVA with the between-subject factor groups (CG, LG, IG, MG) revealed a highly significant effect, *F*(3, 115) = 30.68, *p* < .0001, *η*_*p*_^2^ = .45. Post hoc comparisons (Scheffé) revealed highly significant differences between the control group and all experimental groups (*p* < .01). The line group differed significantly from the intersection group (*p* < .05) and from the maximization group (*p* < .01). There was no difference between the intersection group and the maximization group.

We further assessed the impact of hints, by comparing the increase in intersection strategy after 5 min (calculated as the percentage of solution attempts with intersection strategy after the hint minus before the hint), across groups. A one-way ANOVA with the between-subject factor groups (CG, LG, IG, MG) revealed a highly significant effect, *F*(3, 115) = 18.36, *p* < .0001, *η*_*p*_^2^ = .33. Post hoc comparisons (Scheffé) revealed highly significant differences between the control group and all experimental groups (*p* < .01).

#### Maximization score

**Fig. 5:**
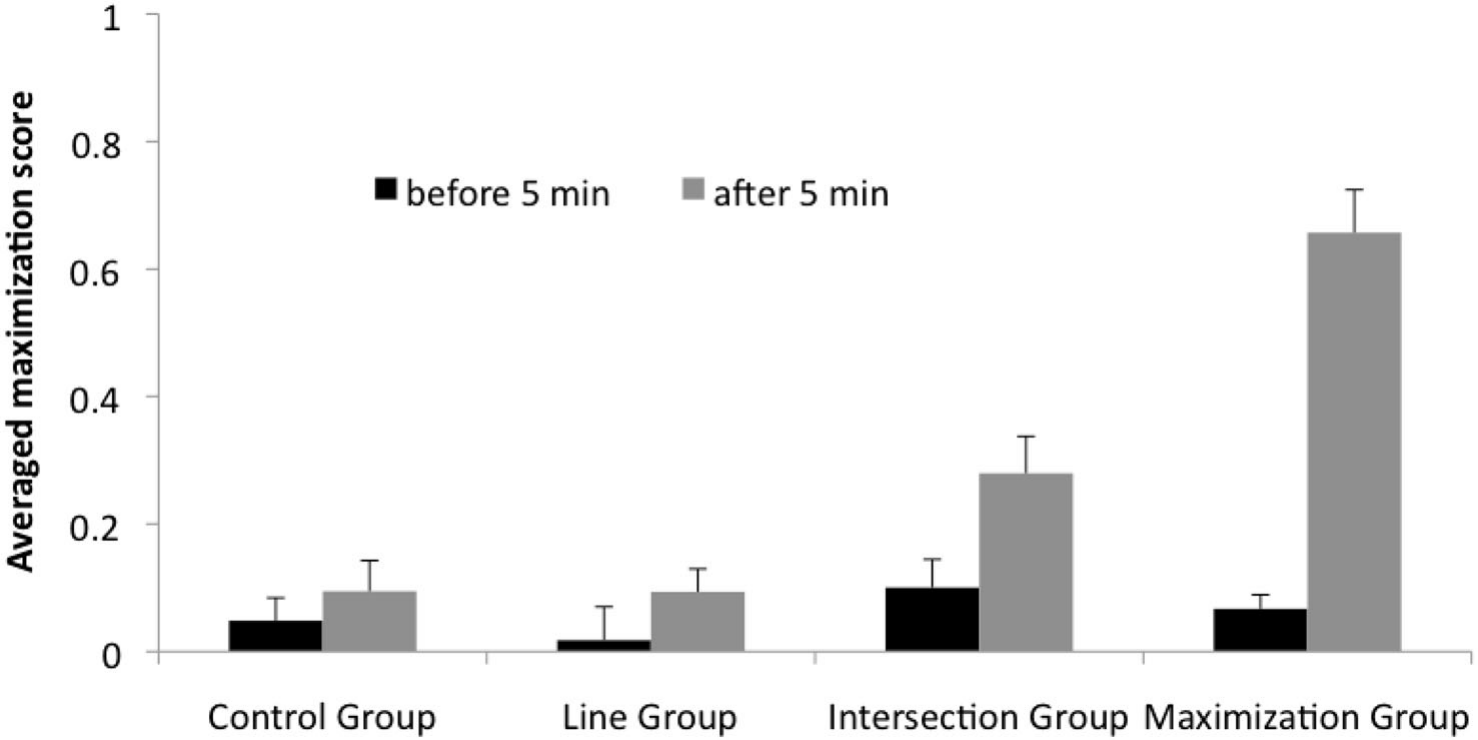
Average maximization score across groups. *Black bars* maximization score before 5 min, *gray bars* maximization score after 5 min. After 5 min, a hint was provided in the experimental groups. The *bars* represent the average value; the *whiskers* represent SE

Figure 5 shows the average maximization score (see “Data analysis” section) for each group. Before the hint, these were positive, but close to zero (Fig. 5 black bars). After the hint, the value in the maximization group showed a pronounced increase and the intersection group showed a somewhat smaller increase (Fig. 5 gray bars). Before the hint, a one-way ANOVA with the factor groups (CG, LG, IG, MG) revealed no significant difference between groups, *F*(3, 115) = .73, *p* > .50, *η*_*p*_^2^ = .02. After the hint, a one-way ANOVA with the factor groups (CG, LG, IG, MG) showed a reliable difference, *F*(3, 115) = 24.89, *p* < .0001, *η*_*p*_^2^ = .40. Post hoc comparisons (Scheffé) indicated that the maximization group differed highly significantly from all other groups (*p* < .01). There was no other significant difference between groups. To rule out the possibility that the effect was caused by the higher amount of solvers in the maximization group, we excluded the solution move from the analysis, since the solution move added a value of +1. A one-way ANOVA revealed a highly significant effect for the factor group, *F*(3, 109) = 12.29, *p* < .0001, *η*_*p*_^2^ = .26. Post hoc tests (Scheffé) revealed significant differences between the maximization group and all other groups (*p* < .01).

To evaluate the amount of increase of maximization due to the hint, we calculated the difference of maximization scores after 5 min minus before 5 min for each group. A one-way ANOVA with the between-subject factor groups (CG, LG, IG, MG) revealed a highly significant effect, *F*(3, 115) = 12.80, *p* < .0001, *η*_*p*_^2^ = .26. Post hoc comparisons (Scheffé) revealed highly significant differences between the maximization group and all other groups (*p* < .01). There were no further significant differences between the groups.

### Hints, solution rate, and variation

#### Solution rate

We compared the solution rate (Table 3) across all groups by pairwise *χ* ^2^ tests. The difference was significant between MG and all other groups: CG and MG, *χ* ^2^(1, 59) = 23.80, *p* < .0001, *Φ* = .64; LG and MG, *χ* ^2^(1, 58) = 26.01, *p* < .0001, *Φ* = .67; IG and MG *χ* ^2^(1, 57) = 14.85, *p* < .0001, *Φ* = .51. These *p* values are considered significant even if we apply the conservative Bonferroni correction for multiple tests (.05/6 = .0083). There was no other significant difference between groups.

**Table 3:** Solution rates across groups

Group	Number of solvers/group size (%)
Control group (CG)	3/30 (10 %)
Line group (LG)	2/29 (7 %)
Intersection group (IG)	6/28 (21 %)
Maximization group (MG)	21/29 (72 %)

#### Variation rate

**Fig. 6:**
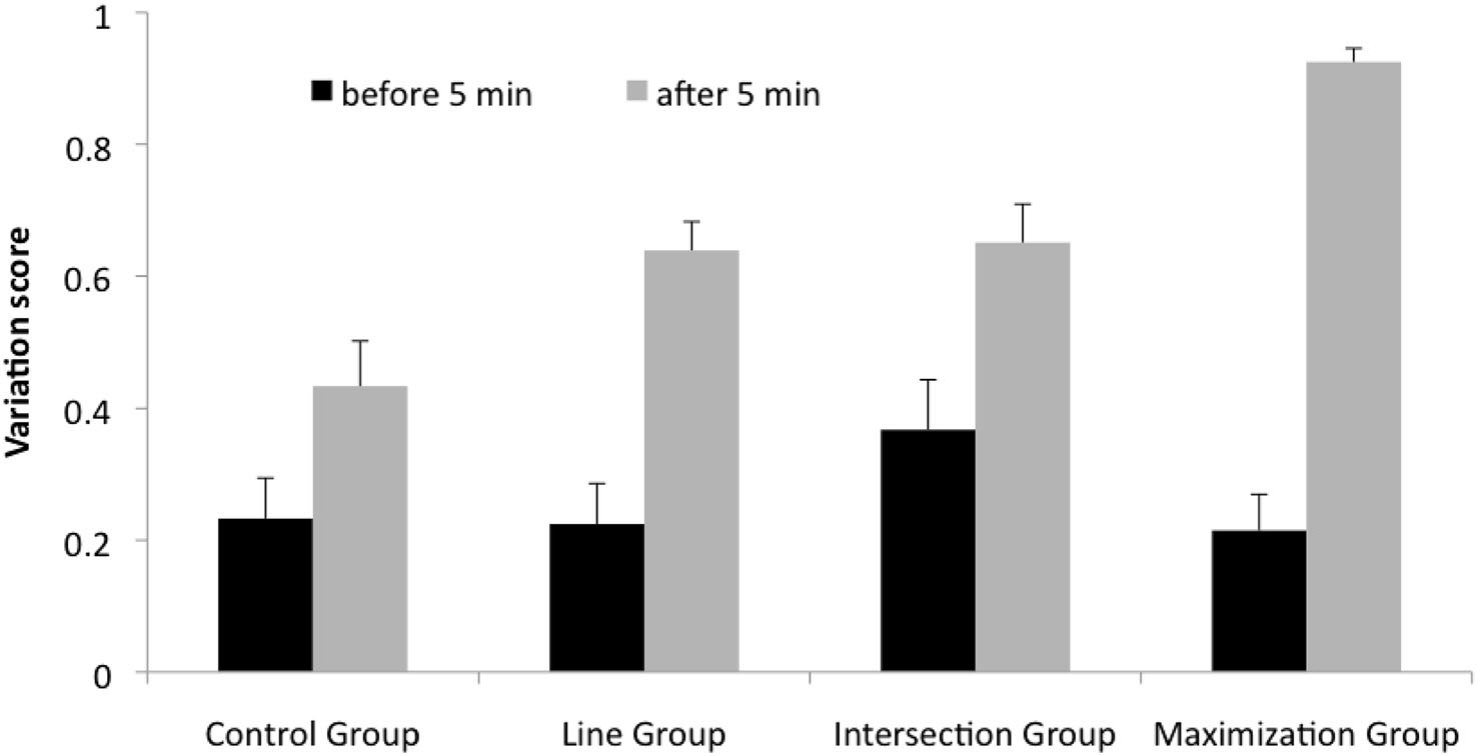
Average variation score across groups. *Black* and *gray bars* variation rate before and after 5 min, respectively. After 5 min, a hint was provided in the experimental groups. The *bars* represent the average value; the *whiskers* represent SE

We tested whether the four groups differed in the variation rate of their solution attempts. We did two separate analyses for the data before 5 min and after 5 min. As Fig. 6 illustrates the variation rate before 5 min was very similar in each group. A one-way ANOVA with the factor groups (CG, LG, IG, MG) revealed no significant difference between the groups, *F*(3, 115) = 1.26, *p* > .30, *η*_*p*_^2^ = .03. After 5 min, the line, intersection, and maximization groups showed a pronounced increase in variation rates (Fig. 6 gray bars). A one-way ANOVA with the factor groups (CG, LG, IG, MG) showed a reliable difference, *F*(3, 115) = 15.78, *p* < .001, *η*_*p*_^2^ = .30. Post hoc comparisons (Scheffé) indicated that the maximization group differed highly significantly from all other groups (*p*s < .01). The intersection group differed significantly form the control group (*p* < .05), and the line group showed a marginal difference from the control group *p* = .05.

To evaluate the amount of increase of variation between groups due to the hint, we computed the difference of variation score after 5 min minus before 5 min for each group. A one-way ANOVA with the between-subject factor groups (CG, LG, IG, MG) revealed a highly significant effect, *F*(3, 115) = 8.57, *p* < .001, *η*_*p*_^2^ = .19. Post hoc comparisons (Scheffé) revealed highly significant differences between the maximization group and the intersection group as well as between the maximization group and the control group (*p* < .01). There were no further significant differences between the groups.

### Solvers and non-solvers

We were interested whether solvers systematically differed from non-solvers, irrespective of group. We compared solvers and non-solvers with respect to the number of participants that made intersections before the hint, and to the variation rate of solution attempts.

#### Intersections

Our prediction was that solvers are more likely to realize the importance of using intersections than non-solvers, even before the hint (MacGregor et al., 2001). In total, there were 32 solvers and 84 non-solvers (see Table 3). We found that 47 % of solvers (15 participants), and 26 % of non-solvers (22 participants) used intersections before 5 min. A *χ* ^2^ test comparing the categories (1 = intersection before hint, 0 = no intersection before hint) between solvers and non-solvers revealed a significant effect, *χ* ^2^(1, 116) = 4.56, *p* < .05, *Φ* = .20, indicating that there is an association between using the intersection strategy in the first 5 min and being a solver. The effect would even be more pronounced, if we included the four solvers that solved the problem without any hint, since all of them used intersections.

#### Variation rate

We tested whether solvers showed a higher variation rate than non-solvers. We found an average variation rate of .43 (SD .24) for non-solvers, and .59 (SD .24) for solvers. An independent groups *t* test (solver, non-solver) revealed a significant main effect, *t*(114) = 3.10, *p* = .01, Cohen’s *d* = .67, indicating a medium effect size.

#### Number of attempts

We tested whether solvers and non-solvers differed in the number of their solution attempts. Non-solvers made an average number of 10.93 attempts (SD 4.91), in contrast solvers made 8.41 (SD 2.76). An independent groups *t* test (solver, non-solver) revealed a significant main effect, *t*(114) = 2.74, *p* = .01, Cohen’s *d* = .62, indicating a medium effect size.

### Binary logistic regression: condition, variation, and maximization

We evaluated the predictive value of the number of solution attempts, maximization scores and variation scores for solving the ten-penny problem. We conducted a binary logistic regression analysis (BLR) (Hosmer, & Lemeshow, 2000). BLR provides a method of analyzing the influence of dichotomous, discrete, or continuous predictors on a binary outcome variable, and has already been successfully applied to the analysis of insight problem solving (Kershaw et al., 2013; Öllinger et al., 2014). BLR produces *B* values, and odds ratios. *B* values indicate the direction of the relationship; odds ratios indicate, for example, the likelihood that a participant in a particular group can be categorized as a solver, e.g., an odds ratio of 2 of a particular group illustrates that a participant of this group is two times more likely to solve the problem than for the baseline (CG) condition (c.f. Öllinger et al., 2014).

We entered two blocks in the model. The first block included the dummy coded predictor groups (CG, LG, IG, MG), the maximization and variation rates after 5 min, and the number of solution attempts. We defined the control group as reference category. The model of this block was significant, *χ* ^2^(6, 115) = 63.51, *p* < .01, and classified 87.8 % of the data correctly. The Nagelkerke *R* ^*2*^ = .61, showing that 61 % of the variability of the data could be explained by the model. The model revealed a significant influence of the two predictor’s maximization after 5 min and variation after 5 min. The first showed an odds ratio of 2.18 and the second an odds ratio of 3.99. In the second block, we controlled for the effect of interactions. We entered all two-way interactions with the predictor groups (maximization after 5 min × group, variation after 5 min × group, and attempts × groups). Although the model of block 2 was significant, *χ* ^2^(15, 115) = 96.26, *p* < .01, there were no significant predictors in this model and the odds ratios of most of the variables were out of range. This might indicate that too many variables were fit into the equation. Therefore, we could not further interpret these results (Table 4).

**Table 4:** Binary logistic regression data modeling the predictive influence of maximization scores, variation rate, and the factor group on solving the problem

Model	*B*	SE	Wald *χ* ^2^	*df*	Sig.	OR	95 % CI	
							Lower	Upper
Block 1								
Group			4.10	3	.25			
LG	−.11	1.08	.01	1	.92	.90	.11	7.42
IG	.169	.94	.03	1	.87	1.17	.19	7.32
MG	1.42	.95	2.23	1	.14	4.13	.64	26.45
Max. after 5 min	.78	.38	4.30	1	.04*	2.18	1.04	4.57
Variation after 5 min	1.38	.56	6.08	1	.01*	3.99	1.33	12.00
Attempts	−.116	.09	1.61	1	.21	.89	.74	1.07

*SE* standard error, *Sig*. significance, *OR* odds ratio, *CI* confidence interval, *LG* line group, *IG* intersection group, *MG* maximization group

* Significant effect

## Discussion

We aimed at investigating the multiple sources of problem difficulty, and the role of search and constraints in a difficult multi-step problem, the ten-penny problem. We hypothesized that a pennies constraint and a separate rows constraint are parts of the problem difficulty. The first constrains participants’ problem representation to use single pennies to solve the problem (and not lines), the second constrains participants’ problem representation to arrange the pennies in separate rows that do not intersect. In three experimental groups, we gradually relaxed these constraints. Additionally, we provided a maximization heuristic that was intended to guide the search process. Accordingly, one group was informed to use lines instead of pennies (line group), a second group received the hint to use intersection of lines to solve the problem (intersection group), and a third group was instructed to maximize the number of intersections (maximization group). The experimental groups were contrasted with a naïve control group, which received no hint. We introduced maximization score and variation rate to investigate differences in search behavior. We aimed at deciphering the concerted interplay of heuristics and representational change when solving a difficult multi-step problem to elaborate on the “special view” of insight problem solving.

### Sources of difficulty

First, we tested whether the hypothesized constraints (pennies constraint and line constraint) and the maximization criterion affected the problem solving process. Analyzing the distribution of zero variation participants (Table 2) showed that before 5 min (no hints) more than half of the participants across all groups used pennies strategy exclusively. This supports our assumption that using pennies and not lines was a constraint from the beginning. Providing hints affected the search for alternative strategies of all experimental groups positively, whereas the control group still contained a high number of participants (33 %) that repeated the pennies strategy throughout the experiment.

All groups significantly differed, except for the intersection group and the maximization group, when we compared them based on the percentage of solution attempts with the intersection strategy after 5 min (CG < LG < IG = MG). This means that all hints effectively increased the probability of using intersections (see Fig. 4).

Comparing groups based on their maximization score after 5 min (Fig. 5 gray bars) showed a different pattern. Only the maximization group differed from the other groups (CG = LG = IG < MG).

### Hints, solution rate, and variation

We tested the effect of hints on problem solving behavior. We compared groups based on their solution rate, and their variation rate. We found that the maximization group performed better than all other groups, with a solution rate of 72 %. Hinting to use lines (without mentioning the number of intersections), or hinting to use intersections (without mentioning the maximization criterion) did not increase solution rates significantly, compared to the control group. The following unexpected rank order for the solution rates was found: CG = LG = IG < MG. This order mirrors the maximization score pattern.

Comparing variation rates after 5 min (Fig. 6 gray bars) across groups revealed a different pattern (CG < LG = IG < MG). When we compared the difference of the variation scores after 5 min minus before 5 min, the maximization group showed a higher difference than the intersection group and the control group, but no other differences were found. While the conclusions from these statistical tests are less clear, it is safe to say, that the hints in the maximization group increased the variation rate more than the other hints.

### Solvers and non-solvers

Looking at strategies within the first 5 min revealed that a higher percentage of solvers (47 %) used intersections before the hint than non-solvers (26 %). The four participants that were excluded from the analyses because they solved the task in less than 5 min also used intersecting lines for the solution. It seems conceivable that solvers overcame self-imposed constraints but had no criterion to restrict the search space at this early stage, and consequently could not monitor the progress of the search process properly.

We found that the variation rate differed significantly between solvers and non-solvers. A lower variation between attempts might be an indicator for the repetition of solution approaches that could be the sign of a self-generated mental set, see zero variation participants in the control group (Beeftink, van Eerde, & Rutte, 2008; Smith, 1995).

Last, we found that the number of attempts differed between solvers and non-solvers. Non-solvers made more attempts, which was expected since they worked on the problem longer. However, the result is not trivial because it also indicates that non-solvers did not quit trying to solve the problem and did not cease problem solving attempts.

#### Binary logistic regression

Modeling the influence of group, variation and maximization after 5 min, and the number of attempts showed that the only significant predictor was variation rate after 5 min and maximization rate after 5 min; that is, higher variation and maximization rates are positively related to the solution of the problem. Although the analyses revealed no significant interaction of any of the chosen predictors with the predictor group it seems plausible to assume that in particular the maximization group that showed the highest maximization and variation rates contribute to this pattern. It is important to note that the hints of the maximization group affected multiple sources of problem difficulty (Kershaw, & Ohlsson, 2004) that helped to increase the variation and maximization rate.

In sum, relaxing the underlying constrains and having the insights to use lines and intersections is a necessary but not sufficient for solving the task. Using lines seemed to reduce the complexity of the task of manipulating ten single pennies (grouping). The hint proved insufficient without finding an appropriate maximization criterion that helps to monitor the progress against the desired goal (MacGregor et al., 2001; Öllinger, Jones, & Knoblich, 2013a, b; Öllinger et al., 2014; Ormerod, MacGregor, & Chronicle, 2002). Interestingly, successful problem solvers were more likely to realize the importance of intersecting lines even before the hint, and also showed a higher variation rate than non-solvers. The importance of variation and maximization was demonstrated by the BLR. A maximization criterion provides a mean to monitor the problem solving progress and to find the goal configuration of the pennies. The last statement could not be fully confirmed by our experimental design, since the maximization group did receive multiple hints (line, intersection, maximization). An open question remains whether providing exclusively a hint about a maximization criterion increases the solution rate. First of all, we think that it is almost impossible to find an appropriate hint that does not relax the underlying constrains at the same time, e.g., “Maximize the number of intersections between the rows of pennies”, or “Maximize the number of pennies that mutually share pennies with other rows” might relax the line and the separate rows constraints too. Even if there was an unambiguous hint, we would expect no effect, just like in the nine-dot problem, where the instruction to draw lines outside the boundary of the given nine dots fails to increase solution rate (see Öllinger et al., 2014). We suggest that further studies need to investigate this claim in more detail.

Surprisingly, even in the maximization group not all participants were able to solve the problem (28 % failed). We predicted that the hints (line + intersections + maximization) would relax all main causes of problem difficulty. This assumption was proven wrong. Either there is still an additional source of difficulty, or the application of the maximization heuristic overstrained some participants (processing factor).

The inefficiency of hints was found in several other insight studies (Chronicle et al., 2001; Kershaw et al., 2013; Öllinger et al., 2014; Weisberg, & Alba, 1981a, b). There is also evidence for the importance of the processing factor for solving multi-step problems. MacGregor et al. (2001) pointed out that a higher look-ahead value (see introduction) is helpful to solve the nine-dot problem, since a higher value allows realizing the violation of a progress monitoring criterion earlier. Importantly, a higher look-ahead value is associated with a higher working memory capacity. In fact, Chein et al. (2010) demonstrated for the nine-dot problem that participants with a higher spatial working memory span drew lines more likely to non-dot points, and benefited effectively from hints. Ash and Wiley (2006) investigated the influence of individual working memory differences on solving insight problems that allowed either multiple moves or only a few moves in a biased problem representation. The authors’ rationale was that only solving the first class of problems should benefit from a higher working memory span, because initially these problems require restricting the search space before a representational change could occur, so that the appropriate search space could be used. Although the authors used problems that had a smaller search space (like the eight-coin problem, Ormerod et al., 2002), we believe that for the ten-penny problem individual working memory differences could play an important role too. It seems plausible to assume that higher variation rates of successful solvers might be closely linked to a higher working memory span, allowing the problem solver to keep track of the history of solution attempts preventing to repeat unsuccessful solution attempts. This ought to be investigated in further studies.

Comparing solvers and non-solvers emphasized the importance of varying solution attempts. Recently, Fedor, Szathmáry, & Öllinger (2015) found that non-solvers repeated their unsuccessful moves more often than solvers in the five-square problem. We suggest that repeating unsuccessful strategies could lead to a self-generated mental set, which in turn, hinders progress; an assumption that is coherent with the early experimental findings on mental set (Birch, & Rabinowitz, 1951; Luchins, & Luchins, 1959; Luchins, 1942). These studies showed that the repeated activation of a successful strategy makes people “blind” for a more efficient or alternative method (see also Bilalić, McLeod, & Gobet, 2008, 2010). Öllinger et al. (2008) showed that the repeated activation of a newly learned insightful solution strategy can even block well-known prior knowledge strategies (see also Chi, & Snyder, 2011). However, in the current study mental set did not result from the repeated activation of a successful strategy, but from the repeated activation of an unsuccessful strategy.

A next reasonable step would be to investigate the expectation and monitoring process of participants when they solve insight problems, and to prevent them from entering a self-generated mental set, instead, increasing the variation of potential solution strategies (Beeftink et al., 2008).

### Implications for insight problem solving

Does it make sense to classify the ten-penny problem as an insight problem? Or is it a problem that could be solved by search (Newell, & Simon, 1972) and should be regarded as nothing special? We opt for the first. Generally, we stated (see Öllinger et al., 2014) that all “classical insight problems” (Dow, & Mayer, 2004; Metcalfe, & Wiebe, 1987; Weisberg, 1995) could be solved with or without insight. The key component that might characterize an insight problem is the problem solvers’ need for a representational change (Fleck, & Weisberg, 2013; Ohlsson, 1992; Öllinger, & Knoblich, 2009). For the ten-penny problem, it seems plausible that there are constraints that have to be relaxed, like using lines or intersections, and this makes it “something special”. As our data show, relaxing constraints and finding new and efficient strategies to restrict the search space was not trivial. It was shown that mainly variation scores after hints predict whether the problem will be solved or not. Taken together the variation data showed that our hints helped to relax the constraints and enhanced the problem solving process.

Our findings could be seen as complementary to the existing evidence about the nine-dot problem (see “Introduction”). This might reconcile the opposing positions of Kaplan and Simon (1990) and Ohlsson (1992, 2011). While the former assumed that insight problems are difficult due to an ill-defined problem representation that results in a much too large search space, the latter suggested that insight problems are difficult (among other things) due to an overconstrained problem representation. We suggest that these assumptions only differ in the importance and function that they attribute to constraints and heuristics in the respective problem.

Typically, in the nine-dot problem, problem solvers start searching in an overconstrained but still large search space, then after repeated failures, they relax the perceptual constraint to attain a search space that allows to make non-dot turns and contains the solution (Kershaw, & Ohlsson, 2004), which in turn has to be restricted by heuristics (Öllinger et al., 2014). As MacGregor et al. (2001) conclusively showed the difficulty was not to find the maximization criterion, but to apply the maximization criterion at the appropriate search space, a process that benefits from higher spatial working memory capacity (Chein et al., 2010).

In contrast, the ten-penny problem requires relaxing constraints to restrict the large search space from the beginning, and a large number of successful solvers realized this need very early. Doing so allows applying the maximization criterion to come up with the solution. Bringing together lines, intersections, and the realization how the number of intersections have to be maximized could be seen as the main insight into the solution of the problem. It might sound paradoxical; but for the ten-penny problem, the relaxation of constraints restricts the search space in contrast to several other studies, where constraint relaxation increases the size of the search space (Ash, & Wiley, 2006; Kershaw et al., 2013; Kershaw, & Ohlsson, 2004; Knoblich et al., 1999; Knoblich, Ohlsson, & Raney, 2001; MacGregor et al., 2001; Öllinger, Jones, Faber, & Knoblich, 2013; Öllinger et al., 2008; Öllinger et al., 2013a, b).

## Conclusions

We think that our study enhances the “special view” by demonstrating the concerted interplay of heuristics and representational change and supports the multiple causes of difficulty account of insight problem solving (Kershaw, & Ohlsson, 2004). Moreover, we think that our study provides a better understanding of the dynamics of insight problem solving at least for multi-step problems. We found that maximization and variation of solution attempts might be beneficial to solve the problem. We propose that higher variation rates help efficient search of the search space and to avoid the repetition of unsuccessful solution attempts. The first might be crucially dependent on a higher working memory capacity (Ash, & Wiley, 2006; Chein et al., 2010) that helps guiding attention to unvisited states of the search space, and at the same time keeping track of the overall problem solving process.

Finally, we propose that our findings about the importance of variation are in harmony with evolutionary accounts of problem solving and creativity (Campbell, 1960; Dietrich, & Haider, 2014; Fernando, Goldstein, & Szathmáry, 2010; Fernando, Szathmáry, & Husbands, 2012; Simonton, 1995). If evolution might be in fact at play at the neuronal level, it is not surprising that a larger variation among candidate hypotheses is crucial for the evolution of the solution.
